# Transformation of Black Phosphorus through Lattice Reconstruction for NIR‐II‐Responsive Cancer Therapy

**DOI:** 10.1002/advs.202305762

**Published:** 2023-12-19

**Authors:** Lie Wu, Mingyang Jiang, Chenchen Chu, Tingting Luo, Yun Hui, Wenhua Zhou, Shengyong Geng, Xue‐Feng Yu

**Affiliations:** ^1^ Shenzhen Key Laboratory of Micro/Nano Biosensing Shenzhen Institutes of Advanced Technology Chinese Academy of Sciences Shenzhen 518055 China

**Keywords:** lattice reconstruction, liposomes, Ni_2_P QDs, NIR‐II photothermal therapy, synergistic therapy

## Abstract

The photothermal performance of black phosphorus (BP) in the near infrared (NIR)‐II bio‐window (1000–1500 nm) is low, which limits its biomedical applications. Herein, ultrasmall nickel phosphide quantum dots (Ni_2_P QDs) are synthesized with BP quantum dots (BPQDs) as the template by topochemical transformation. The size of Ni_2_P QDs is ≈3.5 nm, similar to that of BPQDs, whereas the absorption and photothermal conversion efficiency of Ni_2_P QDs at 1064 nm (43.5%) are significantly improved compared with those of BPQDs. To facilitate in vivo applications, an Ni_2_P QDs‐based liposomal nano‐platform (Ni_2_P‐DOX@Lipo‐cRGD) is designed by incorporation of Ni_2_P QDs and doxorubicin (DOX) into liposomal bilayers and the interior, respectively. The encapsulated DOX is responsively released from liposomes upon 1064‐nm laser irradiation owing to the photothermal effect of Ni_2_P QDs, and the drug release rate and amount are controlled by the light intensity and exposure time. In vivo, experiments show that Ni_2_P‐DOX@Lipo‐cRGD has excellent tumor target capability and biocompatibility, as well as complete tumor ablation through the combination of photothermal therapy and chemotherapy. The work provides a new paradigm for the NIR‐II transformation of nano‐materials and may shed light on the construction of multifunctional nano‐platforms for cancer treatment.

## Graphical abstract

NIR‐I‐responsive black phosphorus quantum dots are topochemically transformed to NIR‐II‐responsive nickel phosphide quantum dots (Ni_2_P QDs) through lattice reconstruction, which are embedded into liposomal bilayers to construct an anti‐cancer nanoplatform (Ni_2_P‐DOX@Lipo‐cRGD). Targeted delivery, on‐demand drug release, and complete tumor ablation are realized by Ni_2_P‐DOX@Lipo‐cRGD.

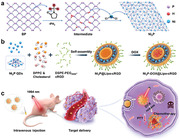

## 1 Introduction

Among the various types of 2D materials, black phosphorus (BP) has been extensively implemented in photothermal therapy (PTT) owing to its broad optical absorption, excellent biosafety and biodegradability, and large specific surface area.^[^ ^1^, ^2^ ^]^ However, after reviewing previous publications regarding BP nano‐material‐mediated PTT, we found that the clinical application of BP as a photothermal agent (PTA) remains limited. First, although intensive studies have focused on improving photothermal conversion efficiency (PCE) by surface modifications, BP‐based nano‐platforms are still responsive to near‐infrared‐I (NIR‐I, 750–1000 nm) light, typically an 808 nm laser.^[^ ^3^ ^]^ The lower tissue penetration depth, maximum permissible exposure (MPE), and spatial resolution of NIR‐I light severely limit the biomedical applications of BP.^[^ ^4^ ^]^ Second, tumors cannot be completely ablated because of the uneven distribution and low accumulation of BP PTAs in tumor cells.^[^ ^5^ ^]^ Third, the ambient instability of BP before reaching a tumor site may adversely affect its photothermal effect, hindering its practical application.^[^ ^6^ ^]^ Therefore, it is of great significance to convert BP into NIR‐II (1000–1500 nm)–activated PTAs with high PCE via structural reconstruction and to further develop an all‐in‐one multifunctional nano‐platform for realizing the targeted delivery of BP and complete removal of tumors.

Recent studies have reported the transformation of BP nano‐materials into NIR‐II‐responsive PTAs. For example, carbon dots responsive to NIR‐II light (NIR‐II‐CDs) were electrostatically adsorbed onto the surface of BP nano‐sheets to form NIR‐II‐CD/BP hybrids.^[^ ^7^ ^]^ More recently, C_60_ was covalently grafted onto BP nano‐sheets using a fullerene covalent passivation method, and the formed BP‐ester‐C_60_ was applied in NIR‐II PTT.^[^ ^8^ ^]^ Despite these BP hybrids exhibiting a considerable NIR‐II PTT effect, the structural stability of the physically or chemically blended strategies raises concerns under complex in vivo environments. Metal phosphides with low‐energy electrons and corresponding NIR‐II absorption may provide a feasible strategy to synthesize NIR‐II‐responsive BP nano‐materials.^[^ ^4^ ^]^ One example is that Fe_2_P nano‐rods (not synthesized from BP) showed a high PCE of 56.6% in the NIR‐II window.^[^ ^9^ ^]^ Other metal phosphides, such as Co_2_P, Ni_12_P_5_, and CoP, have been topochemically transformed from 2D phosphorene sheets for the oxygen evolution reaction.^[^ ^10^, ^11^ ^]^ However, there have been no reports on the transformation of BP nano‐materials for NIR‐II PTT through lattice reconstruction.

Single‐mode PTT often accompanies cancer recurrence owing to the incomplete killing of tumor cells.^[^ ^12^ ^]^ To solve this problem, researchers have developed various combination therapy strategies, such as PTT combined chemotherapy,^[^ ^13^ ^]^ chemo‐dynamic therapy,^[^ ^14^ ^]^ photodynamic therapy,^[^ ^15^ ^]^ radiotherapy,^[^ ^16^ ^]^ gas therapy,^[^ ^17^ ^]^ gene therapy,^[^ ^18^ ^]^ and immunotherapy,^[^ ^19^ ^]^ to improve the therapeutic effect of PTT. Among them, as one of the most widely used and irreplaceable treatment methods, chemotherapy combined with PTT has shown great clinical transformation potential.^[^ ^20^ ^]^ In a previous study, we designed a BP‐liposome nano‐carrier by incorporating BP quantum dots (BPQDs) into liposomal bilayers and encapsulating doxorubicin (DOX) into the interior phase of a liposome.^[^ ^21^ ^]^ The integration of the rapid intracellular release of DOX and local hyperthermia remarkably enhanced cell‐killing efficiency through chemo‐PTT. However, this BP‐liposome composite is a NIR‐I‐responsive nano‐platform; the in vivo therapeutic efficacy has not been evaluated.

**Figure 1:**
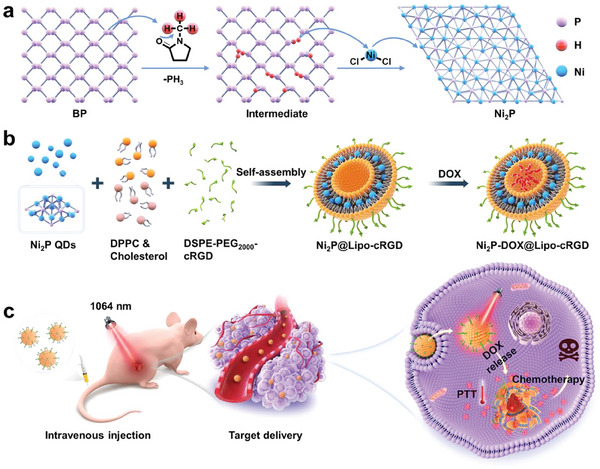
a) Schematic illustration of topochemical synthesis of Ni_2_P QDs from BPQDs templates. b) The preparation process of Ni_2_P‐DOX@Lipo‐cRGD. c) The synergistic effects of Ni_2_P‐DOX@Lipo‐cRGD on efficient ablation of tumors.

Herein, NIR‐I‐responsive BPQDs were topologically transformed into NIR‐II‐responsive Ni_2_P QDs through lattice reconstruction of BP, and the Ni_2_P‐DOX liposomal nano‐platform (Ni_2_P‐DOX@Lipo‐cRGD) was further designed for complete ablation of tumors (**Figure** **1**). The Ni_2_P QDs with a size of 3.5 nm exhibited excellent photothermal performance in the NIR‐II window (1064 nm). The ultrasmall size of Ni_2_P QDs facilitated their insertion into the hydrophobic lipid bilayers, and thus DOX was loaded into the interior aqueous core of the liposome. After targeted delivery to the tumor site, NIR‐II irradiation induced structural cleavage of the liposome and subsequently rapid release of DOX, resulting in complete obliteration of tumors through the combination of PTT and chemotherapy. The NIR‐II transformation of BP may shed light on the construction of other PTAs, and the Ni_2_P‐DOX@Lipo‐cRGD nano‐platform holds tremendous clinical potential for cancer treatment.

## 2 Results and Discussion

### 2.1 Preparation and Characterization of Ni_2_P QDs

**Figure 2:**
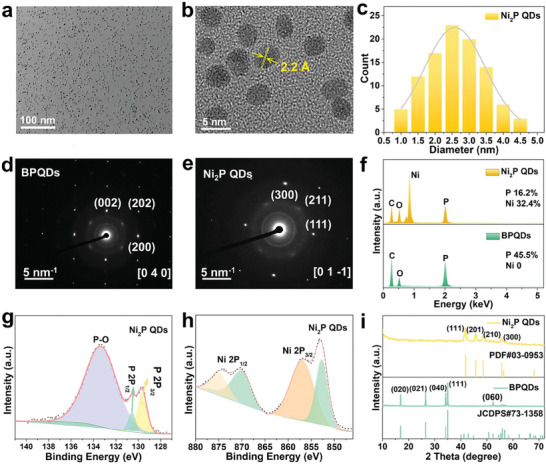
Characterization of Ni_2_P QDs. a) TEM image. b) HR‐TEM image. c) Size distribution measured by DLS. d) SEAD pattern of BPQDs and e) Ni_2_P QDs. f) EDS spectra of BPQDs and Ni_2_P QDs. g) XPS P 2p and h) Ni 2P spectra. i) XRD spectra of BPQDs and Ni_2_P QDs.

BPQDs were prepared by the two‐step ultrasonic liquid exfoliation method previously reported by our team.^[^ ^1^ ^]^ To synthesize Ni_2_P QDs, we sealed the mixture of BPQDs and nickel chloride hexahydrate (NiCl_2_·6H₂O) in N‐methyl pyrrolidone (NMP) solution in a Teflon bottle and heated it to 160 °C under alkaline conditions for 6 h. The yellow dispersion of BPQDs changed to a black solution, and the Ni_2_P QDs were collected by centrifugation. The transmission electron microscopy (TEM) image in **Figure** **2**a shows that the Ni_2_P QDs were dispersed uniformly and had a diameter of ≈3.5 nm. The high‐resolution TEM (HR‐TEM) image in Figure 2b reveals lattice fringes of 2.2 Å, corresponding to the (111) plane of Ni_2_P.^[^ ^22^ ^]^ According to dynamic light scattering (DLS), the particle size of the Ni_2_P QDs water solution distributed from 1 to 5 nm (Figure 2c) corresponded to the diameter measured by TEM, and the zeta potential was −20.4 ± 2.6 mV (Figure S1, Supporting Information). We measured the selected area electron diffraction (SAED) to further study the crystallization state of Ni_2_P QDs. The SAED patterns in Figure 2d,e show distinct diffraction dots from both BPQDs and Ni_2_P QDs, which means that the QDs were single crystals, instead of polycrystals. Unlike BPQDs, the diffraction dots of Ni_2_P QDs could be indexed to the (111), (211), and (300) crystal planes of the hexagonal Ni_2_P QDs phase, so the crystal zone axis was [01‐1]. These results demonstrate that the topochemical reaction not only preserved the QDs morphology but also showed balanced ions migration rates to form the complete crystal domain.

We acquired energy‐dispersive X‐ray spectroscopy (EDS), X‐ray photoelectron spectra (XPS), and X‐ray diffraction spectra (XRD) to further analyze the structure of Ni_2_P QDs. The EDS in Figure 2f shows that in addition to surface‐adsorbed carbon and oxygen, the Ni_2_P QDs showed a nickel‐to‐phosphorus molar ratio (Ni:P) of ≈2:1 but only phosphorus was observed from BPQDs. As shown in Figure 2g, the binding energies at 129.6 and 130.4 eV were assigned to P 2p_1/2_ and P 2p_3/2_ in Ni_2_P QDs, respectively, and the peak at 133.8 eV could be attributed to oxidized P species. The XPS spectrum in the Ni 2p region (Figure 2h) showed two spin‐orbit doublets. The binding energies of 852.6 and 870.2 eV were assigned to Ni ^δ+^ in Ni_2_P.^[^ ^23^ ^]^ The topochemical transformation from BPQDs to Ni_2_P QDs was investigated by XRD (Figure 2i). Compared to the BPQDs (green line), different peaks appeared from Ni_2_P QDs (yellow line) corresponding to the standard XRD pattern JCPDS No. 03–0953.^[^ ^24^ ^]^ These results demonstrate the successful synthesis of Ni_2_P QDs.

The possible mechanism of topochemical transformation is proposed as follows. With a nucleophilic attack from a strong Lewis base, the NMP molecule loses a hydrogen atom and turns into a NMP radical.^[^ ^25^ ^]^ Similarly, phosphorene can donate its unpaired electrons to NMP molecules and remove hydrogen atoms from their methyl groups. Consequently, P atoms are detached in the form of PH_3_. Moreover, the nickel atoms are successfully inserted into the lattice of the BP.^[^ ^26^ ^]^ After the intercalation, the inserted metal atoms are reacted with the phosphorus and are finally transformed into the metal phosphides.

### 2.2 Optical Absorption and Photothermal Properties of Ni_2_P QDs in the NIR‐II Window

**Figure 3:**
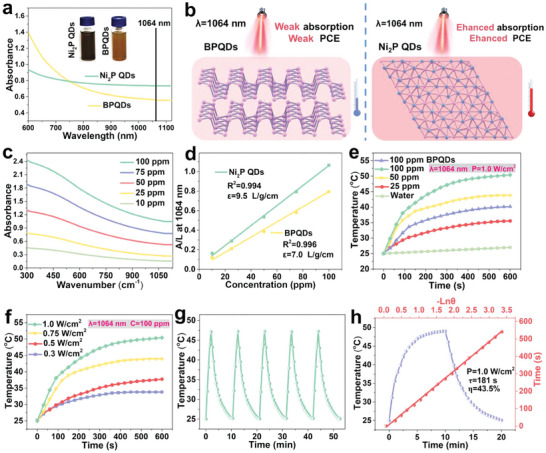
NIR‐II photothermal performance of Ni_2_P QDs. a) Absorption spectra of BPQDs and Ni_2_P QDs dispersed in water at the NIR region. Inset: White‐light photos of BPQDs and Ni_2_P QDs dispersed in water. b) Schematic diagram of absorption of BPQDs and Ni_2_P QDs under NIR‐II light. c) Absorption spectra of Ni_2_P QDs dispersed in water with different concentrations. d) Mass extinction coefficient of BPQDs and Ni_2_P QDs at 1064 nm. e) Photothermal heating curves of Ni_2_P QDs with different concentrations under irradiation with a 1064 nm laser, 1.0 W cm^−2^. f) Photothermal heating curves of Ni_2_P QDs (100 ppm) at different power densities. g) Heating and cooling curves of Ni_2_P QDs for five cycles at 1.0 W cm^−2^. h) Photothermal conversion efficiency of Ni_2_P QDs. Blue line: Photothermal effects of Ni_2_P QDs under 1064 nm laser irradiation for 10 min; Red line: Time constant (*τ*_s_) determined in the cooling period.

The absorption of BPQDs and Ni_2_P QDs in the NIR‐II window was first compared by measuring their UV–vis–NIR spectrum at the same concentration. As shown in **Figure** **3**a, the absorption of BPQDs at 1064 nm was low, resulting in the poor photothermal performance of BPQDs in the NIR‐II window.^[^ ^7^ ^]^ Owing to the presence of low‐energy electrons, the introduction of transition metal ions may enhance the NIR‐II absorption of nano‐materials^[^ ^27^ ^]^. After lattice reconstruction of BPQDs, the Ni_2_P QDs aqueous dispersion had a darker color, and the absorption at 1064 nm was significantly enhanced, which is expected to have the potential of Ni_2_P QDs as NIR‐II PTAs (Figure 3a,b). Ni_2_P QDs have good dispersibility in water owing to their ultrasmall size (1–5 nm) and the negative surface charge (Figure 3a inset). To further evaluate the extinction coefficient, we tested the absorption spectrum of Ni_2_P QDs aqueous dispersion at different concentrations. As shown in Figure 3c, the absorbance of Ni_2_P QDs increased with their concentrations. According to the Lambert–Beer law (*A*/*L* = *𝛼C*, where *𝛼* is the extinction coefficient), a linear relationship was obtained from the plot of *A*/*L* versus *C* and the extinction coefficient of the Ni_2_P QDs was calculated to be 9.5 L g^−1^ cm^−1^ at 1064 nm (Figure 3d), while that of BPQDs was 7.0 L g^−1^ cm^−1^.

Based on the high absorption in the NIR‐II window, the photothermal conversion performance of Ni_2_P QDs with different concentrations (0, 25, 50, and 100 ppm) was investigated by exposing them to a 1064 nm laser with a power density of 1.0 W cm^−2^ (Figure 3e). The temperature increases responded quickly to laser irradiation and were proportional to the power density. Notably, the solution temperature reached 50.4 °C for 100 ppm Ni_2_P QDs, which is much higher than that of the same concentration of BPQDs (only 39.8 °C). The laser power‐dependent photothermal effect (0.3, 0.5, 0.75, and 1.0 W cm^−2^) of Ni_2_P QDs (100 ppm) was subsequently assessed, and the temperature rose as a function of laser power density (Figure 3f). To further evaluate the photothermal stability, we recorded the cycling temperature variations upon 1064 nm laser radiation for 2 min (laser on), followed by natural cooling to room temperature (laser off) for five on/off cycles. As shown in Figure 3g, no significant deterioration was observed during cycling, corroborating the durability of Ni_2_P QDs. The PCE (*𝜂*) of the Ni_2_P QDs was determined to be 43.5% based on the time constant for heat transfer and the maximum steady‐state temperature (Figure 3h). These results indicate that Ni_2_P QDs have excellent optical absorption and photothermal properties in the NIR‐II window.

### 2.3 Preparation and Characterization of Ni_2_P‐DOX@Lipo‐cRGD

Nanoparticles less than 10 nm are excreted instantly before accumulation in a tumor site^[^ ^28^ ^]^. In contrast, single‐mode PTT often leads to tumor recurrence owing to residual tumor cells. Thus, it is necessary to develop a nano‐carrier integrated with the EPR effect, multimodal therapy, and targeted delivery of therapeutic agents for the complete ablation of tumors. In consideration of the clinical translational application, the clinically approved liposome dosage form was selected as a nano‐carrier to transport Ni_2_P QDs and DOX, which were encapsulated in the hydrophobic lipid bilayers and hydrophilic interior phase, respectively. The Ni_2_P‐DOX@Lipo was prepared by a film hydration method previously reported by our group.^[^ ^21^ ^]^ Ni_2_P QDs and lipid materials were dissolved in CHCl_3_ and thus, the ultrasmall Ni_2_P QDs could be inserted into the hydrophobic lipid bilayers after drying using a rotary evaporator. A typical tumor‐targeting molecule, DSPE‐PEG_2000_‐cRGD was further modified on the surface of liposomes (Ni_2_P‐DOX@Lipo‐cRGD, Figure 1b).

**Figure 4:**
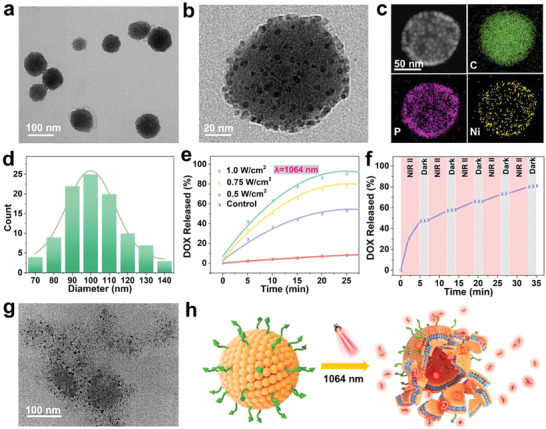
Characterization and NIR‐II photothermal effects of Ni_2_P‐DOX@Lipo‐cRGD. a) TEM image. b) HR‐TEM image. c) HAADF‐STEM image and EDS elemental map of an individual Ni_2_P‐DOX@Lipo‐cRGD nanosphere. d) Size distribution. e) DOX release profiles at different power densities. f) The percent release profile of DOX in the alternating presence of NIR light (1064 nm, 1.0 W cm^−2^) and dark. g) TEM image after NIR irradiation for 20 min. h) Schematic illustration of NIR‐triggered release of DOX. NIR: 1064 nm, 1.0 W cm^−2^.

The TEM image in **Figure** **4**a shows a uniform morphology with a small unilamellar spherical structure and the Ni_2_P QDs were distributed in the liposomes. The HR‐TEM image of a single Ni_2_P‐DOX@Lipo‐cRGD nanoparticle reveals that the Ni_2_P QDs were mainly distributed in the lipid bilayers via hydrophobic self‐assembly with the lipid molecules (Figure 4b). The high‐angle annular dark‐field (HAADF) scanning TEM (STEM) image and EDS mapping revealed the elemental distributions of the single Ni_2_P‐DOX@Lipo‐cRGD nanoparticle (Figure 4c). P and Ni were uniformly distributed in the edge area, confirming that Ni_2_P QDs had been successfully loaded in the outside hydrophobic bilayer of liposomes. As shown in Figure 4d, the mean particle size of Ni_2_P‐DOX@Lipo‐cRGD was ≈100 nm according to DLS. In addition, the encapsulation efficiency of DOX was calculated to be 95.6% by measuring the fluorescence intensity of free and total DOX. As shown in Figures S2–S5 (Supporting Information), the solution color, absorption spectrum, particle size, and drug encapsulation efficiency did not change until the 4th week, indicating the excellent storage stability of Ni_2_P‐DOX@Lipo‐cRGD during cancer therapy.

The photothermal effect and triggered drug release from Ni_2_P‐DOX@Lipo‐cRGD were subsequently evaluated under NIR‐II illumination. Upon exposure to a 1064 nm laser, the temperature of Ni_2_P‐DOX@Lipo‐cRGD rose with the irradiation time and laser power (Figure S6, Supporting Information). As shown in Figure 4e, DOX was released rapidly from liposomes in the first 5 min and underwent a sustained release afterward. After irradiation for 5 min at power densities of 0.5, 0.75, and 1.0 W cm^−2^, the drug release percentages were 21.2%, 30.8%, and 40.5%, respectively, indicating a controlled‐release profile by adjusting laser parameters. Furthermore, the on‐off release behavior was obtained by alternating the presence and absence of NIR light (Figure 4f). The drug release ceased when the light was off, and it restarted when the light was back on, demonstrating an on‐demand drug release. To disclose the mechanism of NIR‐responsive drug release, we observed the morphology of Ni_2_P‐DOX@Lipo‐cRGD by TEM. After irradiation for 20 min, the liposomal structure was disrupted, inducing the release of DOX (Figure 4g). It can be concluded that Ni_2_P‐DOX@Lipo‐cRGD remains stable under physiological conditions and degrades upon laser irradiation (Figure 4h); thus, it exhibits an on‐demand drug release.

### 2.4 In Vitro Anticancer Effects

**Figure 5:**
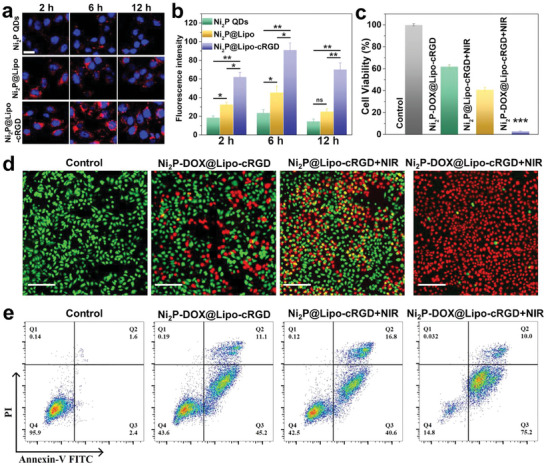
In vitro anti‐cancer effect of Ni_2_P@Lipo‐cRGD with or without DOX loading. a) Confocal fluorescence images and b) fluorescence intensity of MCF‐7 cells after incubation with Cy5.5‐labeled Ni_2_P QDs, Ni_2_P@Lipo, and Ni_2_P@Lipo‐cRGD (*n* = 3). The cell nuclei were stained by DAPI, scale bar is 20 µm. c) Relative cell viabilities (*n* = 6) and d) fluorescence images stained with Calcein‐AM/PI after incubation with PBS (Control group), Ni_2_P‐DOX@Lipo‐cRGD (chemotherapy group), Ni_2_P@Lipo‐cRGD+NIR (photothermal group), and Ni_2_P‐DOX@Lipo‐cRGD+NIR (photothermal‐chemotherapy group) for 24 h. The scale bar is 200 µm. e) Apoptosis assay by Annexin‐V FITC/PI double staining after different treatments for 24 h. Data are presented as mean ± SD. *p* values are calculated by Student's *t*‐test. ^*^ *p* < 0.05, ^**^ *p* < 0.01, ^***^ *p* < 0.001, ns, not significant.

Good biocompatibility is a basic requirement of a drug carrier. Thus, the cytotoxicity of Ni_2_P@Lipo‐cRGD (without loading DOX) was first evacuated by a cell counting kit‐8 (CCK‐8). The cell viability stayed at nearly 100% after incubation for 48 h, even at a high concentration of 100 ppm (the concentration of encapsulated Ni_2_P QDs), thus indicating the outstanding biosafety of Ni_2_P@Lipo‐cRGD (Figure S7, Supporting Information). To verify the uptake of the nano‐carrier, we prepared Cy5.5‐labeled Ni_2_P QDs and loaded them into liposomes. Ni_2_P QDs, Ni_2_P@Lipo, and Ni_2_P@Lipo‐cRGD containing the same concentration of Cy5.5‐labeled Ni_2_P QDs were incubated with cells to study the drug delivery capability of Ni_2_P@Lipo‐cRGD. As shown in **Figure** **5**a,b, the fluorescence intensity of Ni_2_P QDs‐treated cells was relatively weak during the 12 h‐incubation, and Ni_2_P@Lipo‐treated cells exhibited improved fluorescence compared with Ni_2_P QDs‐treated cells. In contrast, for Ni_2_P@Lipo‐cRGD‐treated cells, strong red fluorescence was observed surrounding the blue cell nucleus in the first 2 h, and the fluorescence intensity was much higher than that of the other groups for 12 h. These results indicate that Ni_2_P@Lipo‐cRGD can be efficiently endocytosed and can thus transport more Ni_2_P QDs and other therapeutic agents into cells, owing to the high affinity of cRGD on the surface of the liposome and *α*_v_ *β*_3_ integrin on the cell membrane^[^ ^29^ ^]^.

The combined chemotherapeutic and photothermal effects of Ni_2_P‐DOX@Lipo‐cRGD for anticancer therapy were investigated in vitro. Ni_2_P‐DOX@Lipo‐cRGD or Ni_2_P@Lipo‐cRGD with the same concentration of Ni_2_P QDs (50 ppm) was incubated with MCF‐7 cells for 8 h. After full endocytosis, the nano‐carries were removed, and NIR irradiation was carried out for the PTT groups. The therapeutic effects of different treatment groups were assessed by CCK‐8, cell live/dead co‐staining, and Annexin‐V FITC/PI staining after further incubation for 24 h. Single‐mode therapy induced a cell viability decrease of 61% and 40% in the chemotherapy and PTT group, respectively. In contrast, the cell viability was less than 5% in the combination treatment group (Figure 5c). The live/dead staining also revealed that nearly all of the cells in the photothermal‐chemotherapy group were killed (red fluorescence, Figure 5d). Next, we performed an apoptosis assay by Annexin‐V FITC/PI double staining after different treatments for 24 h. The apoptotic cells (Q2 + Q3) made up as much as 85.2% of the photothermal‐chemotherapy group, which is much higher than that in the single‐chemotherapy group (56.3%) or PTT (57.4%) group (Figure 5e). These results reveal the excellent combined chemo‐photothermal therapy effects of Ni_2_P‐DOX@Lipo‐cRGD on cancer cells.

### 2.5 Targeted Transportation of Ni_2_P QDs into Tumors

**Figure 6:**
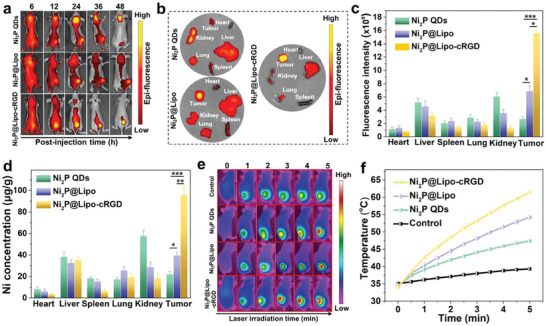
Targeted transportation of Ni_2_P QDs by Ni_2_P@Lipo‐cRGD in MCF‐7 tumor‐bearing mice. a) IVIS fluorescence images after intravenous injection of Cy5.5‐labeled Ni_2_P QDs, Ni_2_P@Lipo, and Ni_2_P@Lipo‐cRGD at different times‐post injection. b) Ex vivo fluorescence images of isolated tissues at 24 h post‐injection. c) Corresponding fluorescence intensity of the isolated tissues calculated by Living Image 4.2 software (*n* = 3). d) Concentrations of Ni element of the isolated tissues determined by ICP‐OES (*n* = 3). e) Infrared thermographic images of mice under NIR irradiation (1064 nm, 1.0 W cm^−2^) 24 h post‐injection of Ni_2_P QDs, Ni_2_P@Lipo and Ni_2_P@Lipo‐cRGD (10 mg kg^−1^). f) Temperature profiles of the tumor sites as a function of irradiation time. Data are presented as mean ± SD. *p* values are calculated by one‐way ANOVA analysis. ^*^ *p* < 0.05, ^**^ *p* < 0.01, ^***^ *p* < 0.001.

The number of therapeutic agents in tumor tissues plays a decisive role in treatment outcomes. In particular, the ultrasmall Ni_2_P QDs are excreted quickly from blood circulation. Thus, the tumor accumulation of Ni_2_P QDs is first assessed before evaluating the in vivo therapeutic effects. Cy5.5‐labeled Ni_2_P QDs, Ni_2_P@Lipo, and Ni_2_P@Lipo‐cRGD were intravenously injected into tumor‐bearing mice at a single dosage of 10 mg kg^−1^. In vivo, fluorescence imaging shows that the fluorescence signals in free Ni_2_P QDs‐treated mice were distributed over the entire mouse and that relatively low fluorescence accumulated in the tumor sites (**Figure** **6**a). Compared to the free Ni_2_P QDs, stronger fluorescence was observed from the tumor site in Ni_2_P@Lipo‐treated mice owing to the passive accumulation of liposomes to tumor sites through the EPR effect. In contrast, for the Ni_2_P@Lipo‐treated mice, the fluorescence signals efficiently targeted and accumulated in tumor sites from 12 h post‐injection (Figure 6a). Ex vivo imaging at 24 h further reveals that fluorescence signals specifically came from tumors in Ni_2_P@Lipo‐cRGD‐treated mice, while free Ni_2_P QDs were mainly accumulated in the kidney because nanoparticles less than 10 nm are metabolized by the kidneys and eliminated from the body (Figure 6b)^[^ ^30^ ^]^. The fluorescence intensity of the tumor in Ni_2_P@Lipo‐cRGD‐treated mice was much higher than that in Ni_2_P@Lipo‐treated mice, suggesting the excellent tumor‐targeting ability of cRGD (Figure 6c).

In addition, the Ni concentration in the main organs and tumor tissues was determined by an inductively coupled plasma‐optical emission spectrometer (ICP‐OES) to directly compare the transportation efficiency of liposomes with or without cRGD modification. The Ni concentration of tumors in Ni_2_P@Lipo‐cRGD‐treated mice was the highest (Figure 6d), consistent with the results of in vivo fluorescent imaging. These results indicate that Ni_2_P QDs can be effectively transported to tumor sites by the Ni_2_P@Lipo‐cRGD, which can be attributed to the EPR effect of liposomes and, especially, the high affinity of cRGD to tumor cells.

Subsequently, the in vivo photothermal properties were tested 24 h post‐injection of Ni_2_P QDs, Ni_2_P@Lipo and Ni_2_P@Lipo‐cRGD. Owing to the high accumulation of Ni_2_P QDs mediated by the cRGD‐liposome, the tumor temperature of Ni_2_P@Lipo‐cRGD‐treated mice increased rapidly to 61.4 °C after 5 min irradiation, which is much higher than that of other groups (Figure 6e and Figure 6f). Therefore, Ni_2_P@Lipo‐cRGD with the active targeting capability is expected to exhibit excellent photothermal effects in vivo.

### 2.6 In Vivo Antitumor Effect

**Figure 7:**
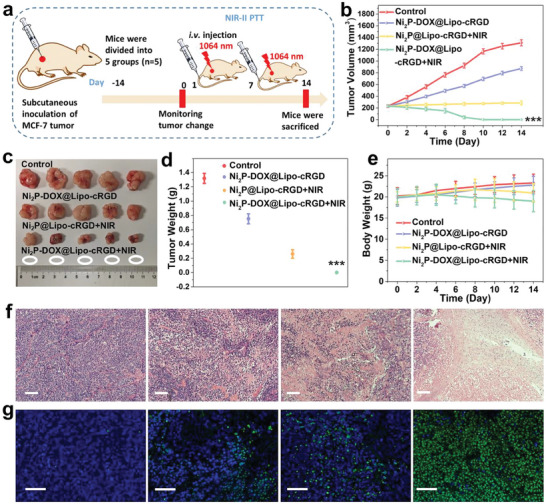
a) Schematic illustration of in vivo antitumor therapy. b) Tumor volume of different groups during the treatment (*n* = 5). (c) Photographs of tumors and d) corresponding tumor weight extracted from the mice at the end of the experiments (*n* = 5). e) Body weight changes (*n* = 5). f) H&E and g) TUNEL of the tumor sections 24 h post‐laser irradiation for each group of mice (Scale bar is 200 µm). Data are presented as mean ± SD. *p* values are calculated by one‐way ANOVA analysis. ^***^ *p* < 0.001.

Based on the targeted delivery of therapeutic agents, the combined chemotherapeutic and photothermal effects of Ni_2_P‐DOX@Lipo‐cRGD were investigated in vivo (**Figure** **7**a). As shown in Figure 7b, tumors in the control group grew rapidly, and the apparent inhibition of tumor growth could be observed from the chemotherapy group. Although single PTT by Ni_2_P@Lipo‐cRGD exhibited an improved antitumor effect, the tumors started growing again after 12 days owing to the incomplete ablation of tumor cells. In contrast, tumors in the photothermal‐chemotherapy group were completely suppressed without recurrence. The photographs of excised tumors after the treatment confirm that the tumors were completely ablated through the combination of PTT and chemotherapy mediated by Ni_2_P‐DOX@Lipo‐cRGD (Figure 7c,d). Furthermore, no body weight change was observed during the treatment process (Figure 7e), and no organ damage (Figure S8, Supporting Information) was observed at the end of treatment, corroborating the excellent biosafety of Ni_2_P‐DOX@Lipo‐cRGD.

The tumors were collected on day 2 and examined by immunohistochemistry to demonstrate the antitumor mechanisms. As shown in Figure 7f,g, the tumor tissues of the photothermal‐chemotherapy group exhibited more severe nucleus shrinkage and plasmatorrhexis (H&E staining) and a larger degree of cell apoptosis (green fluorescence) compared with the other groups. These results show that the Ni_2_P‐DOX@Lipo‐cRGD has strong clinical potential in multimodal cancer therapy.

## 3 Conclusion

In summary, NIR‐I‐responsive BPQDs were topochemically transformed into NIR‐II‐activated Ni_2_P QDs, which were further embedded into liposomal bilayers to fabricate a multifunctional nano‐platform for complete tumor ablation. The Ni_2_P QDs exhibited excellent NIR‐II‐responsive photothermal performance upon exposure to a 1064 nm laser, yielding a PCE of 43.5%. Based on the photothermal property of Ni_2_P QDs, the on‐demand release of DOX from the liposome interior can be well regulated. In addition, the in vitro and in vivo experiments indicate that Ni_2_P‐DOX@Lipo has good anticancer effects and that the combined chemo‐photothermal therapy induces the complete ablation of tumors. Thus, our work provides a new paradigm for the design of a multimodal cancer treatment strategy.

## Conflict of Interest

The authors declare no conflict of interest.

## Supporting information

Supporting Information

## Data Availability

The data that support the findings of this study are available from the corresponding author upon reasonable request.
