# Supplementary material for: Transformation of Black Phosphorus through Lattice Reconstruction for NIR‐II‐Responsive Cancer Therapy
Source: Adv Sci (Weinh). 2023 Dec 19;11(3):2305762. doi: 10.1002/advs.202305762 (PMC10797469; doi:10.1002/advs.202305762)
Supplement: Supplementary file 1 — Supporting Information [file ADVS-11-2305762-s001.pdf]

ADVANCED SCIENCE
Open Access

Supporting Information

for *Adv. Sci.*, DOI 10.1002/advs.202305762

Transformation of Black Phosphorus through Lattice Reconstruction for NIR-II-Responsive Cancer Therapy

Lie Wu, Mingyang Jiang, Chenchen Chu, Tingting Luo, Yun Hui, Wenhua Zhou, Shengyong Geng* and Xue-Feng Yu*

Supporting Information

Transformation of black phosphorus through lattice reconstruction for NIR-II-responsive cancer therapy

Lie Wu, Mingyang Jiang, Chenchen Chu, Tingting Luo, Yun Hui, Wenhua Zhou*, Shengyong Geng*, and Xue-Feng Yu

Dr. L. Wu, Dr. M. Jiang, C. Chu, Dr. T. Luo, Dr. Y. Hui, Prof. W. Zhou, Prof. S. Geng, Prof. X.-F. Yu

Shenzhen Key Laboratory of Micro/Nano Biosensing, Shenzhen Institutes of Advanced Technology, Chinese Academy of Sciences, Shenzhen 518055, China

E-mail: wh.zhou@siat.ac.cn; sy.geng@siat.ac.cn

Experimental Section

Materials: The black phosphorus (BP) crystals were obtained from Mophos (www.Mophos.cn) and stored in a dark Ar glove box. Nickel chloride hexahydrate ($NiCl_2 \cdot 6H_2O$) (99.99%), N-Methyl-2-pyrrolidone (NMP, 99%), doxorubicin (DOX), potassium methylate, trichloromethane ($CHCl_3$) and ethanol were purchased from Aladdin Reagents. Dipalmitoyl-phosphatidylcholine (DPPC) and cholesterol were acquired from Sigma-Aldrich. DSPE-PEG_{2000}-cRGD and Cy5.5-NH_2 were bought from Ruixi Biological Technology Co., Ltd (Xi'an, China). DMEM media, fetal bovine serum, PBS, trypsin were purchased from Gibco Life Technologies (AG, Switzerland). 4',6-diamidino-2-phenylindole (DAPI) staining solution, Cell Counting Kit-8 (CCK-8), 4% parafoemaldehyde (PFA), Calcein-AM, and propidium iodide (PI) were bought from Beyotime (Shanghai, China). All the chemicals were analytical reagent grade and used without further purification.

Synthesis of BP quantum dots (BPQDs): The BPQDs was synthesized by simple liquid exfoliation described previously [1]. Briefly, 30 mg of the BP crystals were added to 30 mL NMP followed by sonication with a sonic tip of 19-25 KHz for 12 h (2s with the interval of 4 s) at the power of 1200 W. The dispersion was sonicated in an ice bath for another 12 h at 300 W. The resulting dispersion was centrifuged at 9000 rpm for 15 min and the supernatant containing BPQDs were decanted gently.

Synthesis of nickel phosphide quantum dots (Ni_2P QDs): The BPQDs (2 mg), 10 mg of $NiCl_2 \cdot 6H_2O$, 1 mg of potassium methylate were dispersed in 15 mL of NMP, stirred, and

transferred to a Teflon bottle. The bottle was sealed and heated in an oven. After reacting for 6 h at 160 °C, the mixture was centrifuged at 9000 rpm for 10 min to collect the precipitate of Ni_2P QDs which were then rinsed with ethanol three times.

Synthesis of Ni_2P QDs-based liposomal nanoplatform (Ni_2P@Lipo-cRGD): The Ni_2P QDs (0.1 mmol), DPPC (0.6 mmol), cholesterol (0.3 mmol), and DSPE-PEG_{2000}-cRGD (0.05 mmol) were dissolved in $CHCl_3$ and dried by a rotary evaporator at reduced pressure at 37 °C to produce a thin film. The film was further dried under vacuum 4 h to ensure complete solvent removal. The lipid film was then hydrated with PBS and sonicated by a VCX 130 probe sonicator for 15 min (40 s on and 10 s off in each cycle). After incubation at 60 °C for 1 h, the centrifugation at 9000 rpm was performed to remove the unencapsulated Ni_2P QDs.

Encapsulation of DOX into Ni_2P@Lipo-cRGD (Ni_2P-DOX@Lipo-cRGD): DOX was encapsulated into liposomes by the transmembrane pH gradient-driven encapsulation technique (inside acidic) as described by our group [2]. A Sephadex G-50 column eluted with PBS (pH 7.4) was used to separate the unencapsulated drug. The loading efficiency was calculated by fluorescence spectrophotometry (λ_{ex} = 505 nm, λ_{em} = 559 nm, Hitachi F-4600, Japan) according to the following equation: Encapsulation efficiency (%) = 100 × $(I_{max}-I_o)/I_{max}$, where I_o is the fluorescence intensity of the liposome suspension at the initial time and I_{max} is the fluorescence intensity after the addition of 0.5% Triton X-100.

Characterizations: Transmission electron microscopy (TEM) and energy dispersive

spectroscopy (EDS) was conducted on the JEM-3200FS (JEOL, Japan) at an acceleration voltage of 200 kV. X-ray photoelectron spectroscopy (XPS) was conducted on the Thermo Fisher ESCALAB 250Xi XPS. X-ray diffraction (XRD) was conducted on the SmartLab X-ray diffractometer (Rigaku, Japan). The size distribution and zeta potential were determined by dynamic light scattering (DLS) using the Zetasizer 3000 HAS (Malvern Instruments Ltd., UK). The UV-Vis-NIR absorption spectra were acquired on an ultraviolet-visible spectrophotometer (U-3900, Hitachi, Japan).

Photothermal effects: The Ni_2P QDs dispersed in water with different concentrations (0, 25, 50, 100 ppm) were exposed to a 1064 nm laser with a power density of 1.0 W/cm^2. Additionally, 100 ppm Ni_2P QDs aqueous solutions were irradiated with the 1064 nm laser with different power densities (0.3, 0.5, 0.75 and 1.0 W/cm^2). The laser spot was adjusted to cover the entire surface of the sample and an infrared thermal imaging camera (Fluke Ti27, USA) was used to monitor the temperature change including the rise and natural cooling.

Calculation of the extinction coefficient (ε): ε was calculated using the following equation: A = εbc. A is the absorbance of Ni_2P QDs at 1064 nm, b is the diameter of the quartz test tube, c is the concentration of Ni_2P QDs.

Calculation of the photothermal conversion efficiency of Ni_2P QDs: The photothermal conversion efficiency (η) was calculated by equations 1-4.

$$\eta = (hS(T_{\max}-T_{\mathrm{surr}})-Q_{\mathrm{dis}})/I(1-10^{-A}) \quad (1)$$

$$hS = \sum mC_p/\tau_S \quad (2)$$

$$\tau_S = - t/\ln\theta \quad (3)$$

$$\theta = (T - T_{surr})/(T_{max} - T_{surr}) \quad (4)$$

where h is the heat transfer coefficient, S is the surface area of the container, τ_S is the time constant for heat transfer from the system, m is mass of products, C_p is specific heat capacity of solvent, T_{max} is the equilibrium temperature of Ni_2P QDs, T_{surr} is the ambient temperature of the surroundings, I is the laser power density, and A is the absorbance of Ni_2P QDs at 1064 nm.

NIR-light-controlled drug release: One milliliter of Ni_2P-DOX@Lipo was placed in a quartz cuvette with a 1 cm path length and each sample was irradiated with the 0.5, 0.75 and 1.0 W/cm^2 1064 nm laser, respectively. The amount of DOX released (%) from liposomes was calculated by the following equation: DOX released (%) = 100 × $(I_t-I_o)/(I_{max}-I_o)$, where I_o is the fluorescence intensity of the sample at the initial time, I_{max} is the fluorescence intensity after the addition of 0.5% Triton X-100, and I_t is the measured fluorescence intensity at various time intervals.

Cell culture: The MCF-7 cell lines were purchased from China type culture collection (CTCC) through the American Type Culture Collection (ATCC). MCF-7 cells were cultured in DMEM medium supplemented with 10% (v/v) fetal bovine serum and 1% (v/v) penicillin/streptomycin under humidified conditions of 5% CO_2 at 37 °C.

In vitro toxicity assay: The MCF-7 cells were seeded on 96-well plates at a density of 5

$\times 10^4$/mL and cultured overnight. The culture medium was replaced with a medium containing serial dilutions of Ni_2P@Lipo-cRGD (0, 20, 40, 60, 80 and 100 ppm of loaded Ni_2P QDs). After incubation for 48 h, the medium was removed and a medium containing the 10% (v/v) CCK-8 solution was added to each well followed by incubation for 2 h. A microplate reader (FilterMax F5, Molecular Devices, USA) was used to measure the absorbance at 450 nm. The untreated cells were represented as control group.

Intracellular uptake: The positively charged Cy5.5-NH_2 with the concentration of 0.1 mg/mL was added and mixed with the negatively charged Ni_2P QDs solution. Subsequent sonication for 1 h and stirring for another 12 h at room temperature were carried out in the dark. The obtained sample was fully washed with water until fluorescence of Cy5.5 cannot be detected in the centrifugation supernatant.

The MCF-7 cells were seeded on confocal dishes at a density of 5×10^4/mL and cultured for 24 h. The culture medium was removed, and a medium containing Cy5.5-labeled Ni_2P QDs, Ni_2P@Lipo, and Ni_2P@Lipo-cRGD with a concentration of 20 ppm Ni_2P QDs was added to each dish. After incubation for 2, 6, and 12 h, the cells were washed and fixed. The cell nucleus was stained by DAPI with the concentration of 100 ng/mL for 10 min. Fluorescent images were taken on a confocal microscope (Leica TCS SP5).

In vitro antitumor efficiency: The MCF-7 cells were seeded on a 96-well plate at a density of 5×10^4/mL and cultured for 24 h. The culture medium was replaced by a medium containing Ni_2P@Lipo-cRGD or Ni_2P-DOX@Lipo-cRGD. For the antitumor assay, the cells were treated

with PBS (Control group), Ni_2P-DOX@Lipo-cRGD (chemotherapy group), Ni_2P@Lipo-cRGD+NIR (photothermal group), and Ni_2P-DOX@Lipo-cRGD+NIR (photothermal-chemotherapy group). After incubation for 4 h, the cells in the photothermal group and photothermal-chemotherapy group were illuminated with the 1064-nm laser (1.0 W/cm^2) for 5 min. The cells in Control group were also irradiated for 5 min to determine the cytotoxicity of NIR light. The cells were incubated for another 24 h and the cell viability of each group was determined by the CCK-8. In the live/dead assay, the medium was removed and Calcein-AM (5 μg/mL) and PI (5 μg/mL) were added. After incubation for 15 min, the cells were washed with PBS, and fluorescent images were captured by a fluorescence microscope (IX71, Olympus, Japan).

Animal and xenograft tumor models: The study was approved by the Administrative Committee on Animal Research of the Shenzhen Institutes of Advanced Technology, Chinese Academy of Sciences. The approval number for animal experiments is SIAT-IACUC-20211126-CLS-CLJMZX-PT-A0722-01. The Balb/c nude mice (female, 4–6 weeks old) were purchased from Charles River Laboratory Animal Technology Co., Ltd. (Beijing, China) and raised in an SPF animal laboratory. MCF-7 cells (1×10^7) in 100 μL of PBS were injected subcutaneously into the right back side of mice. The tumor volume was observed and measured every 2 days, and the tumor size was calculated according to the following formula: volume (V) = (tumor length) × (tumor width)^2/2.

Targeted transportation of Ni_2P QDs into tumors: Following respectively intravenous

injection of Cy5.5-labeled Ni_2P QDs, Ni_2P@Lipo and Ni_2P@Lipo-cRGD (10 mg/kg), the fluorescence signals of the Cy5.5 from whole body were detected using an IVIS Spectrum (Caliper IVIS Spectrum, PerkinElmer, USA) at designed time point (6, 12, 24, 36, 48 h). The mice were sacrificed at 24 h after administration for *ex vivo* fluorescence distribution in tumors and major organs (heart, liver, spleen, lung, and kidney). All images were normalized and analyzed by Living Image 4.2.

A piece of each tissue (0.2 g) was weighed, digested in HNO_3 (65%) overnight, and then concentrations of Ni were determined by an inductively coupled plasma-optical emission spectrometer (ICP-OES, 7000DV, PerkinElmer, USA).

Photothermal effects of the tumor site: At 24 h post-injection of Ni_2P@Lipo or Ni_2P@Lipo-cRGD, the mice were anaesthetized and the entire region of the tumor was irradiated by the 1064 nm NIR laser (1.0 W/cm^2) for 5 min. The temperature of the tumors under laser irradiation were recorded by an infrared thermal imaging camera (Ti27, Fluke, USA).

In vivo anticancer effects: The tumor-bearing mice were randomly assigned to four groups (n = 5 per group): PBS (Control group), Ni_2P-DOX@Lipo-cRGD (chemotherapy group), Ni_2P@Lipo-cRGD+NIR (photothermal group), and Ni_2P-DOX@Lipo-cRGD+NIR (photothermal-chemotherapy group). For the intravenous injection experiments, 100 μL of the above indicated solution was injected *via* the tail vein once a week. At day 1 post-injection, the photothermal and photothermal-chemotherapy groups were anesthetized and specifically

irradiated by a 1064-nm continuous wave laser (1.0 W/cm^2) for 5 min. The changes in the tumor volume and body weight were recorded every 2 days. At day 14, all of the mice were sacrificed, and the tissues, including those of the heart, liver, spleen, lung, kidney and tumor of the mice were harvested for histopathological, immunohistochemical, and TUNEL analyses according to standard laboratory procedure. In brief, the tissues were fixed in 4% PFA, embedded in paraffin, sliced to 5 μm in thickness, and stained with hematoxylin and eosin (H&E). The apoptosis of tumor cells in the tissue was investigated using the TUNEL method according to the manufacturer's instructions (Jiancheng Bioengineering institute, Nanjing, China). All of the stained tissue sections were observed and imaged with a microscope (Olympus BX53, Japan).

Statistical analysis: Data were presented as mean ± SD as specified and the statistical significance was calculated by the Student *t*-test or one-way ANOVA analysis. $^{*}p < 0.05$, $^{**}p < 0.01$, and $^{***}p < 0.001$ were defined as statistical significances, ns, not significant. Statistical analysis was performed using SPSS Statistics 24.0.

Supplementary Figures

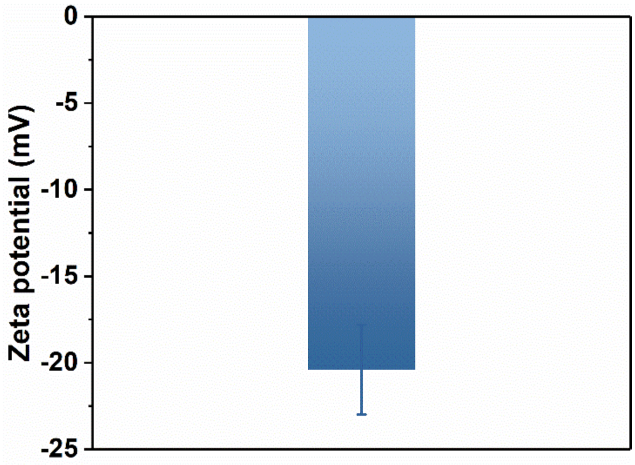

Figure S1. Zeta potential of Ni_2P QDs water solution (n = 3).

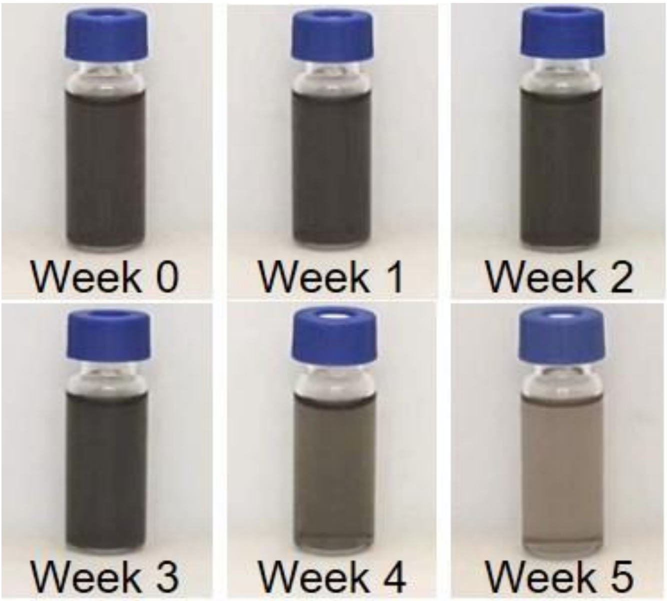

Figure S2. White-light photos of Ni_2P-DOX@Lipo-cRGD after storing at room temperature for different periods of time.

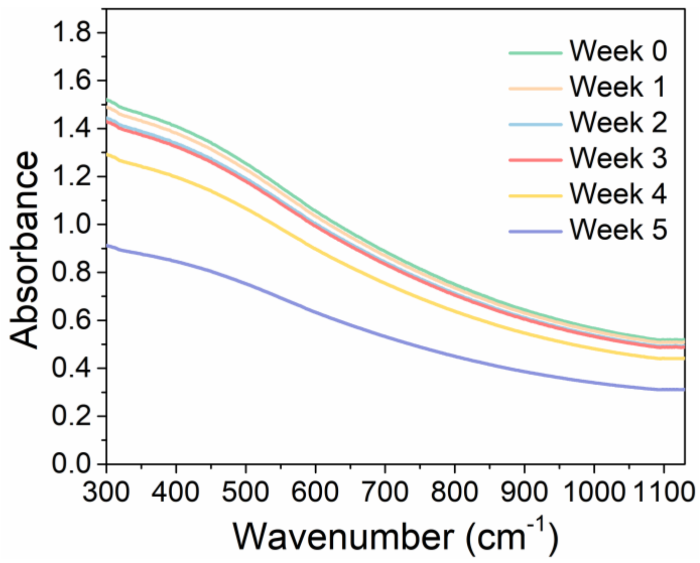

Figure S3. Absorption spectra of Ni_2P-DOX@Lipo-cRGD after storing at room temperature for different periods of time.

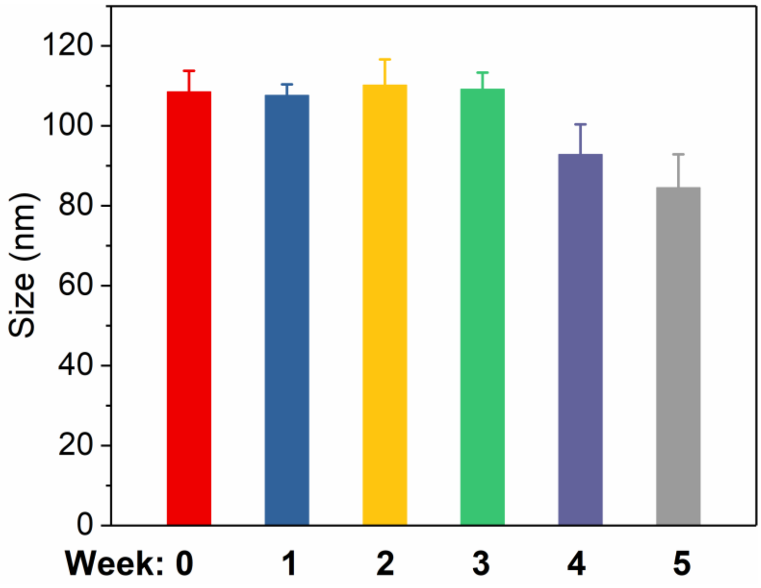

Figure S4. Changes in particle size of Ni_2P-DOX@Lipo-cRGD after storing at room temperature for different periods of time (n = 3).

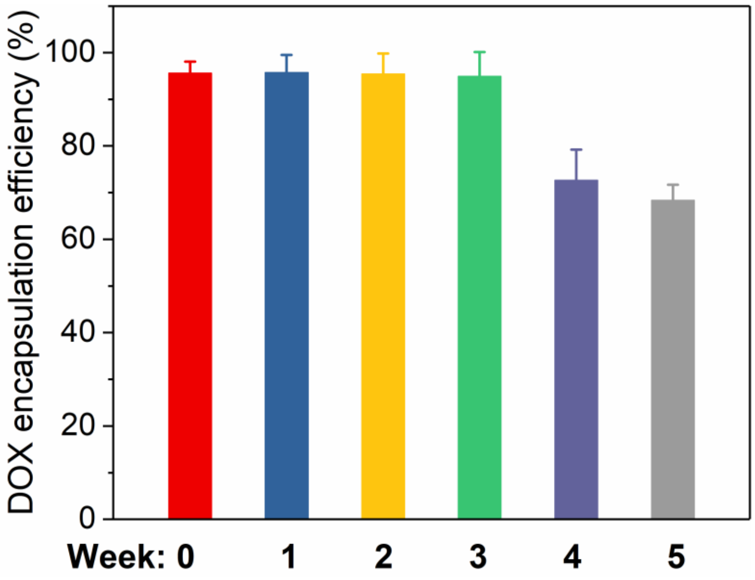

Figure S5. Changes in drug encapsulation efficiency of Ni_2P-DOX@Lipo-cRGD after storing at room temperature for different periods of time (n = 3).

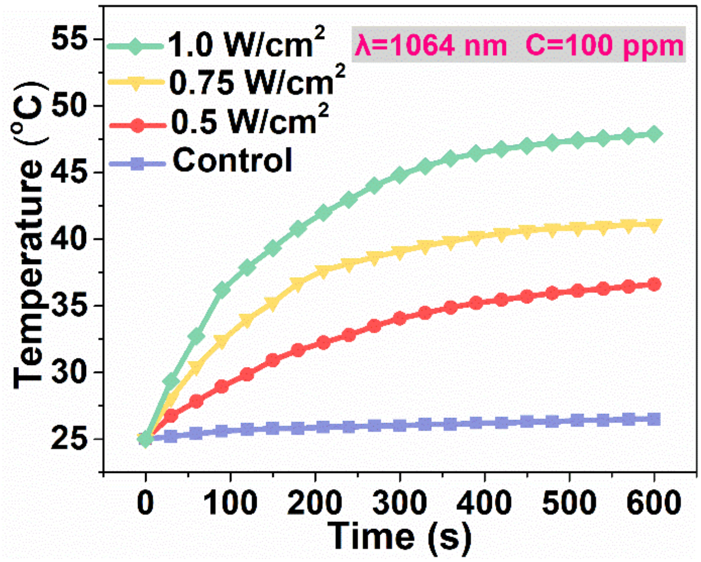

Figure S6. Photothermal heating curves of Ni_2P-DOX@Lipo-RGD (100 ppm of Ni_2P QDs) at different power densities.

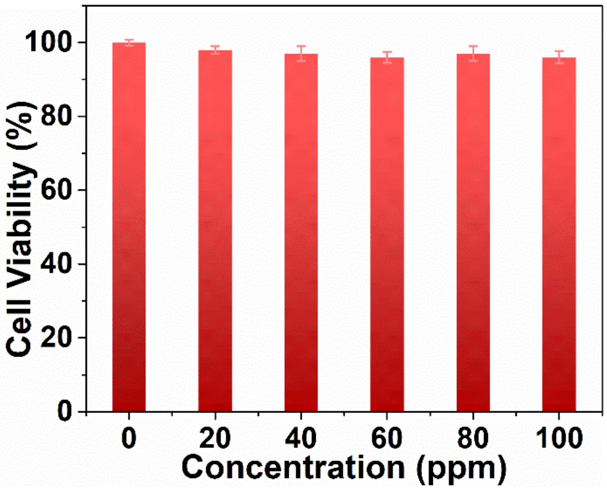

Figure S7. Cell viability of the MCF-7 cells after incubation with Ni_2P@Lipo-cRGD with different concentrations of Ni_2P QDs for 48 h (n = 6).

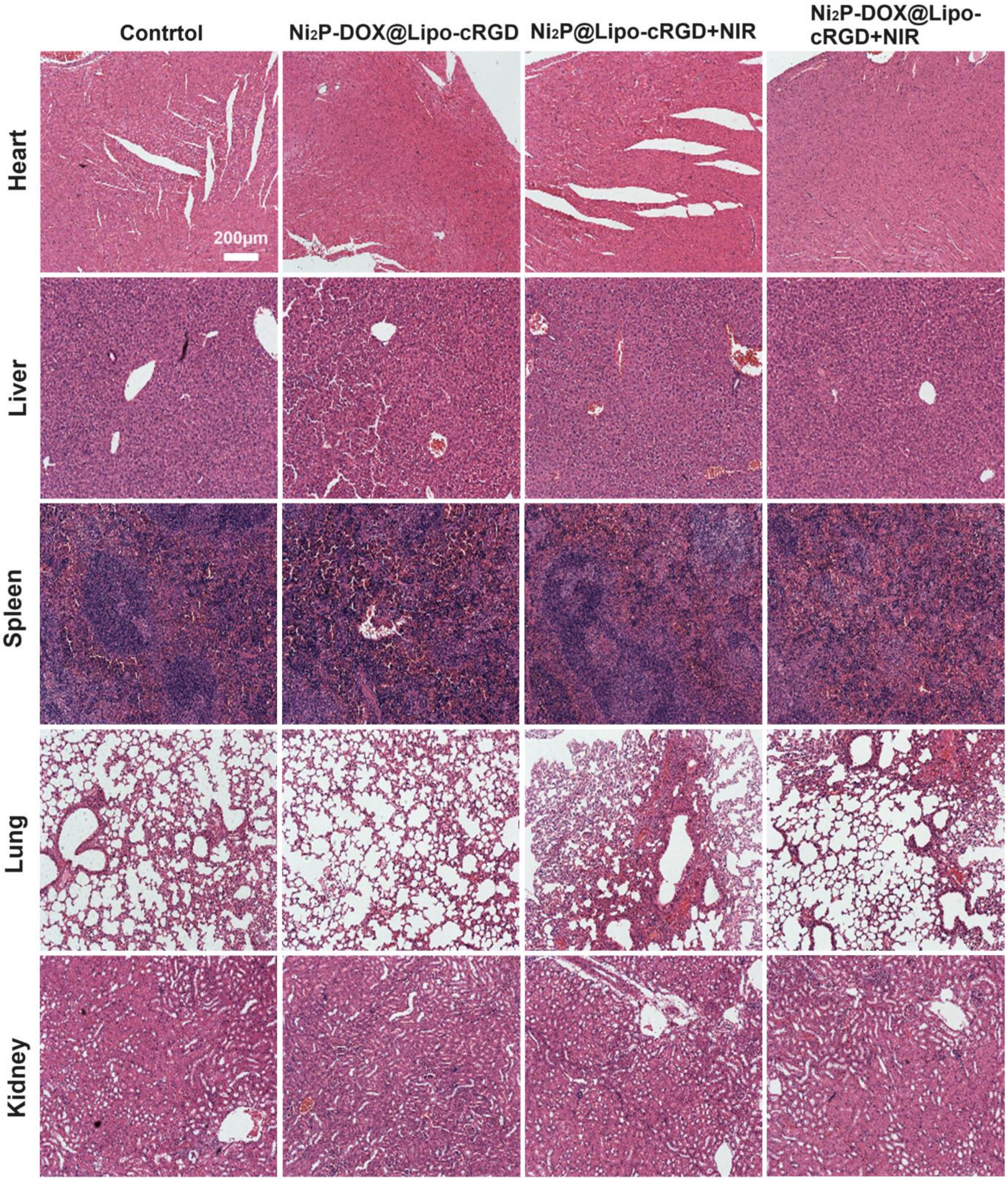

Figure S8. H&E staining images obtained from the main organs after treatments.

References

[1] Z. Sun, H. Xie, S. Tang, X. F. Yu, Z. Guo, J. Shao, H. Zhang, H. Huang, H. Wang, P. K.

Chu, *Angew. Chem., Int. Ed.* **2015**, *54*, 11526;

[2] S. Geng, L. Wu, H. Cui, W. Tan, T. Chen, P. K. Chu, X. F. Yu, *Chem. Commun.* **2018**, *54*, 6060.
